# Interconnected effects of witnessing household violence, food insecurity, and mental health among peri-urban Cape Town youth: A mediation analysis

**DOI:** 10.2989/17280583.2025.2579960

**Published:** 2026-01-02

**Authors:** Miriam Hartmann, Marie C.D Stoner, Danielle Giovenco, Anna Mia Ekström, Abigail M Hatcher, Yanga Zembe Zondi, Nontembeko Qwabe, Audrey Pettifor, Linda-Gail Bekker, Anna E Kågesten

**Affiliations:** 1 Department of Global Public Health, Karolinska Institutet, Stockholm, Sweden; 2 Women’s Global Health Imperative, Research Triangle Institute International, Berkeley, California; 3 Desmond Tutu HIV Centre, University of Cape Town, Cape Town, South Africa; 4 Department of Infectious Diseases, South General Hospital, Stockholm, Sweden; 5 Gillings School of Global Public Health, University of North Carolina at Chapel Hill, Chapel Hill, United States; 6 School of Public Health, Faculty of Health Sciences, University of the Witwatersrand, Johannesburg, South Africa; 7 School of Built Environment and Development Studies, University of KwaZulu-Natal, Durban, South Africa

**Keywords:** domestic violence, food insecurity, mental health, sub-Saharan Africa, mediation analysis

## Abstract

**Background:**

In sub-Saharan Africa, young people face high burdens of food insecurity and sexual and gender-based violence (SGBV), both linked to poor mental health.

**Objective:**

This study tested whether food insecurity predicts common mental disorders through household SGBV, and conversely, whether SGBV predicts common mental disorders through food insecurity.

**Method:**

Data were drawn from 534 participants aged 13 to 25 years in the longitudinal Bidirectional, Upbeat communication and Differentiated Distanced care for Young people (BUDDY) cohort study in Cape Town (2021–2022), surveyed at baseline, three months follow-up, and six months follow-up.

**Results:**

At six months follow-up, 24% reported common mental disorder symptoms, 52% had experienced food insecurity, and 33% had witnessed household SGBV. When food insecurity was modelled as the primary exposure, it was not associated with common mental disorders at six months follow-up (OR = 1.00; *p* = 0.99), and SGBV did not mediate this relationship (Average Direct Effect (ADE) = −0.05; Average Causal Mediated Effect (ACME) = 0.02). In contrast, when witnessing SGBV was the exposure, it was strongly associated with common mental disorders (OR = 2.19; *p* = 0.006). Food insecurity at three months follow-up was more common among those exposed to SGBV at baseline (OR = 1.55; 95% CI [1.06–2.27]; *p* = 0.024). Mediation analysis indicated most of SGBV’s effect on common mental disorders was direct (ADE = 0.17; 95% CI 0.05–0.30), with only a small proportion mediated by food insecurity (5%).

**Conclusion:**

Findings were contrary to our primary hypothesis that food insecurity would drive common mental disorders via SGBV. Instead, household SGBV emerged as the stronger predictor of common mental disorders, with food insecurity playing a modest role. Preventing household violence may be critical to improving youth mental health, while research should further examine food insecurity’s mental health impacts across contexts and timeframes.

## Introduction

Common mental disorders (CMD) are a leading cause of morbidity and mortality, particularly among young people (Guthold et al., 2021). In 2021, an estimated 79 million young people aged 15 to 24 years, including 38.5 million aged 15 to 19 and 40.7 million aged 20 to 24 years, experienced symptoms consistent with CMD, globally (Liu et al., 2025). While many of these youth are from low- and middle-income countries, too few psychological and psychiatric resources are available to treat symptoms of CMD in such contexts (Alonso et al., 2018; Thornicroft et al., 2017). Thus, programs targeting other drivers of mental health urgently require exploration (World Health Organization, 2022). Two potential targets are food insecurity and exposure to sexual and gender-based violence (SGBV). Both have robust evidence associating their experience with CMD (Oram et al., 2022; Pourmotabbed et al., 2020). However, less is known about how SGBV and food insecurity, which are closely related, operate with one another to influence CMD.

Globally, food insecurity has a strong association with symptoms of CMD (Elgar et al., 2021; Pourmotabbed et al., 2020), including among youth in low- and middle-income countries (Elgar et al., 2021; Porter et al., 2022; Trudell et al., 2021). Much of this research is cross-sectional, but it theorises that food insecurity precedes and contributes to poor mental health through behavioural and biological mechanisms including stress and depression (Pourmotabbed et al., 2020). Indeed, qualitative research has found that the experience of food insecurity can lead to stress and create a sense of separation from others, including stigma, shame, and depression (Ayano et al., 2020). Building on this, quantitative and qualitative research from sub-Saharan Africa found that social support could minimise the relationship between food insecurity and CMD (Burgess & Campbell, 2014; Natamba et al., 2017; Tsai et al., 2012). Given the potential influence of food insecurity on CMD through social disconnection, it may not be surprising that SGBV is also associated with food insecurity.

Experiencing or witnessing SGBV, which represents an extreme form of social disconnection, has similarly been associated with stress and poor mental health among young people as well as adults (Gibbs, Dunkle, et al., 2018; Gibbs, Jewkes, et al., 2018; Machisa et al., 2017; Mannell et al., 2022; Marçal, 2022; Meinck et al., 2015; Neigh et al., 2009; Nicodimos et al., 2010; Stern, 2023). Attention to its relationship with food insecurity has been more recent and, while relatively small, is growing. A 2022 meta-analysis identified 38 studies exploring the association between food insecurity and SGBV, finding that food-insecure women had twice the odds of experiencing SGBV than food-secure women. Despite this strong link, most studies were cross-sectional, limiting our understanding of directionality, and most were with adult women (Hatcher et al., 2019). Like food insecurity’s association with CMD, the dominant theory is that it precedes SGBV. In South Africa, research during COVID-19, a time when both food insecurity and social disconnection may have been heightened, reported that youth faced economic hardship and food insecurity (Jesson et al., 2024). Additionally, during COVID-19, food insecurity was identified as a key driver of stress and anxiety in households (Duby et al., 2022), and as a driver of violence, along with broader economic hardship (Mahlangu et al., 2022; Zharima et al., 2024).

## Goal of the study

In the present study, we aimed to conduct a mediation analysis to examine the relationship between food insecurity and later CMD via the mediating role of SGBV exposure, among young people in peri-urban Cape Town, South Africa. This study is meant to build on our baseline findings that witnessing household SGBV was the most dominant form of SGBV reported by youth, and that food insecurity and CMD were significantly associated with this exposure (Hartmann et al., 2023). Our primary hypothesis was that the youth experience of food insecurity simultaneously impacted their household members, contributing to SGBV, and eventually impacting the youth’s CMD. As a secondary hypothesis, we explored whether witnessing SGBV was associated with CMD and if this relationship was mediated by food insecurity, given that a household member’s victimisation may impact that individual’s efficacy to obtain food for the household, thus mediating the negative impact on youth mental health status.

## Methods

### Study design and context

This study represents a secondary analysis of longitudinal data from the Bidirectional, Upbeat communication and Differentiated Distanced care for Young people (BUDDY) cohort study. The BUDDY study is described in more depth elsewhere(Giovenco et al., 2024; Hartmann et al., 2023). However, in brief, it had two aims:

- To examine how COVID-19 lockdown orders impacted young people living with and without HIV (YPLWH vs. YPLWoH) in terms of their experiences of SGBV; and
- To test, through a randomised control trial, the feasibility and acceptability of a remote antiretroviral treatment delivery system among YPLWH.

The study was conducted in Cape Town, in two townships within the Cape Flats area, an area known to have high levels of violence, linked back to its original development as a location for the forced removal of people of colour during apartheid (van der Westhuizen & Gawulayo, 2021).

The study period overlapped with the COVID-19 pandemic lockdowns in South Africa, including the very strict lockdown period from 26 March to 1 May 2020. At this time, the government mandated that people could only leave their homes for essential needs like buying food or attending medical appointments, and there was a complete prohibition on alcohol sales. Some relaxation in measures began in early May, allowing people to be outside within a 5km radius of their residence from 06h00 to 09h00 (Haider et al., 2020). However, full relaxation of the restrictions was not seen until January 2022. Notably, these COVID-19 measures significantly affected South Africa’s economy and heightened individual economic vulnerability, a factor amplifying exposure to violence and compounding challenges like food insecurity (Arndt et al., 2020). A large proportion of South Africans, particularly women, work in the informal economy with no employment contracts and no benefits, which leaves them even more vulnerable to loss of income than those employed in the formal sector. Reports indicate that women in this sector experienced a 70% decrease in income in May and June 2020, just after the lockdown. While the government implemented a social intervention to supplement lost income, this applied only to the formal sector (Saloshni & Nithiseelan, 2022).

### Participants and procedures

Participants in the BUDDY study were young people aged between 13 and 24, living in the designated study region and not intending to relocate for at least half a year. They were required to have consistent access to a mobile phone capable of receiving an SMS, either their own or through a guardian, especially for the preliminary randomised controlled trial and subsequent follow-ups. Recruitment methods included distributing flyers, street-level engagement, and direct recruitment at a public clinic or via phone, based on clinic data. The enrolment spanned from February to October 2021, with a six-month follow-up period. Participants completed a baseline survey, administered by interviewers, that covered their sociodemographic attributes, encompassing aspects like food insecurity, experiences of SGBV within their homes, and CMD linked to the COVID-19 situation. More detailed variables can be found in the referenced work by Hartmann et al (2023). Follow-ups took place at the third and sixth months, with surveys either over the phone or face-to-face. Data collection utilised the secure REDcap platform (Harris et al., 2019). The aim was to have a total sample of 600 to identify a 10% shift in SGBV incidents, based on an initial SGBV prevalence of 30% and 80% statistical power. A total of 535 respondents participated in the baseline visit, 364 of these completed the three-month follow-up (68% response rate), and 311 completed the six-month follow-up (58%). Participants not retained were those unable to be contacted at follow-up.

### Measures

#### Dependent variable

CMD at six months was the primary outcome for this secondary analysis and was measured using two validated screening tools. CMDs broadly include conditions such as depression, anxiety, and somatic symptom disorders (Goldberg & Huxley, 1992; Risal, 2012). In this study, we operationalised CMDs by measuring symptoms of depression and anxiety, as these are among the most prevalent and impactful mental health conditions in our population of interest (Santomauro et al., 2021). While we acknowledge that CMDs encompass a wider range of disorders, our focus on depression and anxiety aligns with prior research in similar contexts and reflects key concerns related to violence exposure and mental health (Oram et al., 2022; Oram et al., 2017; Stansfeld et al., 2017). These tools capture self-reported symptoms of depression and anxiety.

The 9-item Patient Health Questionnaire (PHQ-9; range = 0–27; *α* = 0.89) captured probable depression (Kroenke et al., 2001), which was dichotomised (0 = no, 1 = yes) to demonstrate the presence of any mild+ depression, defined as a score of 5 and above, or any moderate+ depression, defined as a score of 10 and above.

The 2-item Generalized Anxiety Disorder (GAD-2; 0–6; *α* = 0.71) was used to capture self-reported symptoms of probable anxiety. Scores were also dichotomised to indicate possible anxiety disorder (0 = no, 1 = yes) (Bhana et al., 2019), such that scores of 3 and above represent the clinical anxiety cutoff. Probable mild+ depression and anxiety were further combined into the binary variable probable mild+ common mental disorders. The same was done for probably moderate+ depression and anxiety to create the binary variable probable moderate+ common mental disorder (Goldberg & Huxley, 1992).

#### Independent variables

The binary exposure variable of witnessing SGBV among household members, hereafter referred to as SGBV for simplicity, was measured at baseline and three months follow-up. This was determined through three questions that individually queried the occurrence of physical (e.g., instances of pushing, kicking, or slapping), verbal, or psychological (e.g., acts of humiliation or explicit threats), and instances of sexual (e.g., coercion into non-consensual intercourse) forms of violence within the household setting. At baseline, participants were asked about the occurrence of these behaviours since the COVID-19 lockdown and at three months, in the past three months. These questions represent a condensed version of validated survey measures on SGBV victimisation utilised by the World Health Organization (García-Moreno et al., 2005), which are often used due to time and resource constraints limiting the use of the full 25 to 30-item scale (Feldhaus et al., 1997; Roberts et al., 2023; Sherin et al., 1998). Responding “yes” to any of these items was coded as “Any SGBV”.

Food security, also a binary exposure variable asked at baseline and three months, was measured using a single item asking participants whether they felt worried about having enough food in the past month (0 = no, 1 = yes). This represents one item on the Household Food Insecurity Access Scale (Coates et al., 2007).

#### Covariates

Covariates, theorised a priori based on a review of the literature, included other risk factors for SGBV or food insecurity measured at baseline: age, gender, HIV status, race, language, intimate partnership status during lockdown, hazardous alcohol use during the past year using the 3-item Alcohol Use Disorders Identification Test (AUDIT-C; 0–12; *α* = 0.82) (Bush et al., 1998; Morojele et al., 2017), household type (formal or informal), school status and employment status. Participants were also asked whether they received a food parcel during lockdown (0 = no, 1 = yes). We combined school and employment status into a binary measure referred to as “not in education, employment or training” (Social Exclusion Unit, 1999), representing participants who were not in school or employed. Participants who reported being in a relationship were also asked about their exposure to intimate partner violence (IPV), measured using the World Health Organization Violence Against Women Instrument (García-Moreno et al., 2005). This instrument investigates distinct experiences related to controlling behaviours, as well as emotional, physical, and sexually violent actions. An extra query was integrated concerning reproductive coercion, addressing situations where participants faced pressure or were subjected to unprotected intercourse by a partner with the intent of pregnancy. A combined measure of all forms, e.g. “Any IPV”, was created (0 = no, 1 = yes). A Directed Acyclic Graph was developed using DAGitty (Textor et al., 2011) to identify the covariates that may confound the relationship between the primary variables of interest (i.e., food insecurity, SGBV, and CMD).

### Statistical analysis

A series of *t*-tests for continuous variables (e.g., age) and chi-square tests for binary and categorical variables were conducted to characterise the overall study population and to explore the baseline distribution in covariates by household SGBV exposure. Race and language were not included, considering the limited variability observed among the study sample (< 98% identified as black African and isiXhosa speaking). Prevalence estimates were calculated for the primary outcome (binary mild+ CMD variable at six months), exposures (food insecurity or SGBV variable at baseline), mediators (SGBV or food insecurity at three months), and covariates (other baseline risk factors).

**Figure 1.**
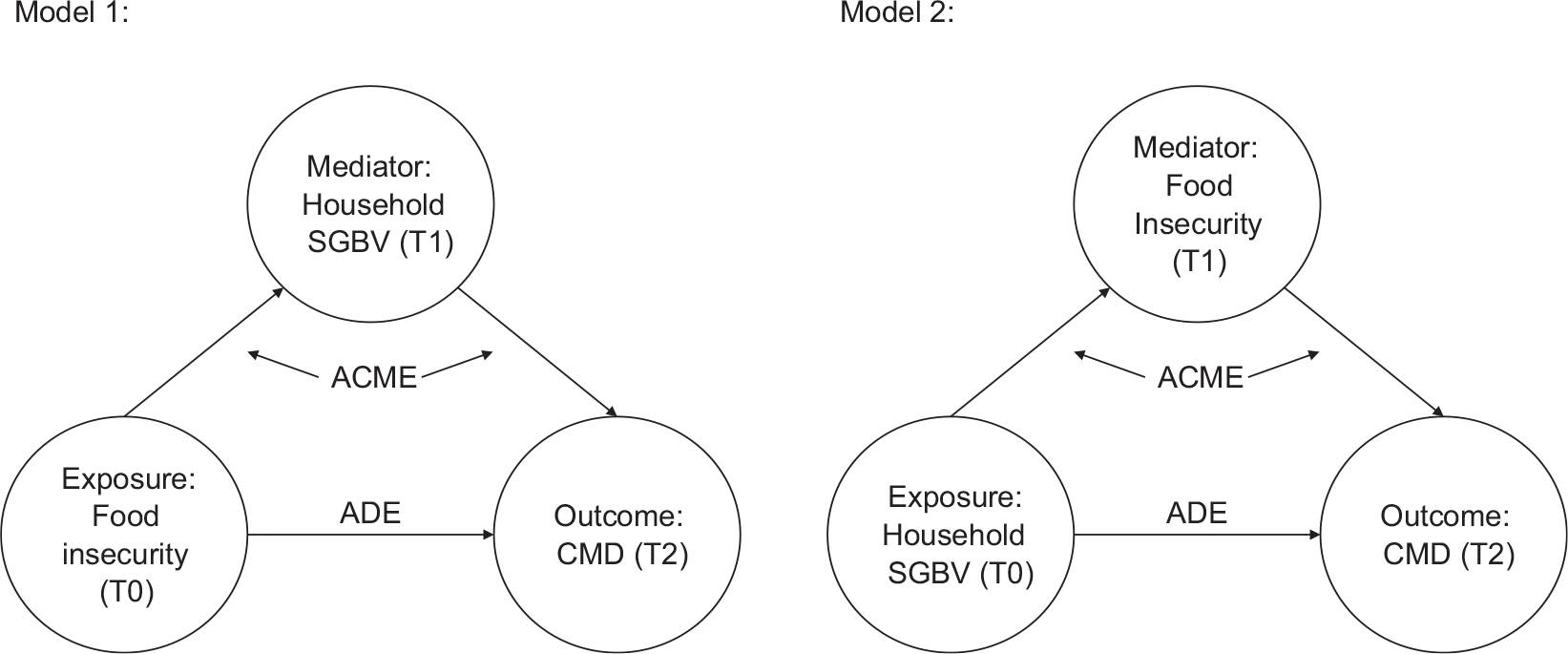
Conceptual models for mediation analysis *Note*. ACME = average causal mediated effect; ADE = average direct effect; SGBV = sexual and gender-based violence; CMD = common mental disorder

A mediation analysis using the Stata command medeff (Valeri, 2023), in STATA 17 (StataCorp, 2021), was conducted to test the direct (not through the mediator, i.e. through other pathways related to SGBV or food insecurity) and indirect (through SGBV or food insecurity) associations between SGBV as the exposure and mild+ CMD as the outcome. As a sensitivity analysis, we also ran the same models using moderate+ CMD as the outcome. Medeff offers a simulation-based approach to mediation analysis, such that it simulates the potential values of the mediator and the outcome values given the simulated mediator values. Mediation models suggest that a mediator falls in the pathway between the exposure and the outcome (Nguyen et al., 2021; VanderWeele & Tchetgen Tchetgen, 2017). These models aim to identify the average causal mediated effect (ACME), which represents the effect of the exposure on the outcome through the indirect pathway of the mediator and inclusive of predictive covariates, and the average direct effect (ADE) of an exposure on an outcome not through the mediator. See Figure 1 for our two models.

First, the association between the exposure (SGBV or food security) and outcome (CMD) was tested using a probit model, a maximum-likelihood model appropriate for binary outcome variables. Next, a probit model was used to examine the association between the binary mediator (food security or SGBV) and the binary CMD outcome measure. The mediation analysis then identified the ACME and the ADE of an exposure under an assumption of sequential ignorability, which holds that the relationship between the exposure and the mediator cannot be explained by unmeasured confounders (Hicks & Tingley, 2012).

Unadjusted and adjusted analyses were conducted. In adjusted models, we controlled for age, gender, HIV status, household type (formal or informal), intimate partnership status during lockdown, “not in education, employment or training” status, exposure to IPV, and hazardous alcohol use. Confounders were selected based on our Directed Acyclic Graphs for each exposure-outcome relationship. An interaction term reflecting potential interactions between the exposure and mediating variable was also included. All mediation models additionally controlled for baseline values of both the mediator and the outcome to account for pre-existing levels and to strengthen causal interpretation(Roth & MacKinnon, 2013; VanderWeele & Tchetgen Tchetgen, 2017). As a sensitivity analysis, we also re-estimated mediation models restricting the sample to participants without the mediator at baseline (food insecurity or SGBV, depending on the model).

Given that 45% of the sample was retained at all three visits, inverse probability weighting was used to account for potential differential loss to follow-up by exposure status. The denominator of the weights was estimated using the predicted probability from logistic models for loss to follow-up. Models included the exposure of food insecurity to account for differential loss to follow-up over time and all baseline characteristics hypothesised to relate to unequal participation. Inverse probability weights were then calculated as the inverse of the predicted probability of loss to follow up for all participants. We compared characteristics of the retained sample with weighting to the unweighted overall population at baseline to make sure that they were similar. We also examined regression coefficients and the distribution of weights to ensure that there were no extreme values. The weights were then included in the final mediation model for the outcome to account for loss to follow up such that we upweighted the individuals who remained in the sample to represent those who were lost to follow-up.

### Ethical considerations

The research received approval from both the University of Cape Town Human Research Ethics Committee (HREC REF:448/2020) and the Swedish Ethical Review Authority (EPN Dnr 2020–04903). Before participation, all individuals provided either written informed consent or assent, especially those below 18 years of age. Minors had the discretion to seek or waive parental approval, with IRB approval, recognising the sensitive themes of the research, which encompassed topics of violence and sexual as well as reproductive health. Protocols adhering to South Africa’s Children’s Act of 2005 (South African Government, n.d.) were followed for handling and reporting disclosures of violence. Any participant indicating exposure to violence was provided with an opportunity to consult with a study counsellor; this was a requisite for those below 18 years. Cases substantiated as abuse were subsequently referred to a social worker for continued support. Moreover, throughout the research duration, the team vigilantly monitored potential social harms, ensuring prompt detection and intervention for any abuses stemming from the study.

## Results

### Sample characteristics

At baseline, participants had a mean age of 19 years (SD = 3 years). Most were female (70%; *n* = 368), living without HIV (60.5%; *n* = 318), and living in formal dwellings (62.5%; *n* = 328) with an average of four other people. Approximately half of the participants had a partner during lockdown, and just over 40% (*n* = 211) were not in school or employed. Half of participants reported food insecurity in the past month (52.2%; *n* = 273), close to half reported symptoms of probable mild+ CMD (47.3%; *n* = 245), and one-third reported witnessing any household SGBV since the initial COVID-19 lockdown (33.1%; *n* = 165). As indicated in Table 1, participants who reported food insecurity at baseline were more likely to be older (*p* < 0.001), living without HIV (71.2%, *p* < 0.001), had an intimate partner during lockdown (55.4%, *p* = 0.01), not employed or in school (39.5%, *p* < 0.001), to report symptoms of probable mild+ CMD (52.3%, *p* = 0.025), and to have been exposed to SGBV in their household (40%, *p* < 0.001) compared to those not reporting food insecurity. Participants who witnessed SGBV in the household at baseline were significantly more likely to be living without HIV (70.3%; *p* = 0.006), report food insecurity (63.0%, *p* < 0.001) and to report symptoms of probable mild+ CMD (65.5%; *p* < 0.001) or moderate+ CMD (33.9%; *p* < 0.001) compared to those who had not witnessed SGBV. Finally, 42% (*n* = 148) of participants were food insecure at three months, and 24% (*n* = 74) experienced symptoms of probable mild+ CMD at six months. Participants who remained in the study tended to be slightly older (OR 1.08; *p* = 0.05), to be living without HIV (OR: 2.09; *p* = 0.001), to have received a food parcel during COVID-19 lockdown (OR 1.71; *p* = 0.01), and to not be employed or in school (OR 1.68; *p* = 0.04). Inverse probability weighting successfully weighted the sample. See supplementary Table S1 for a comparison of baseline characteristics between the total unweighted sample and the weighted sample completing all study visits.

### Model 1: Mediating role of witnessing household SGBV in the association between food insecurity and CMD

In the adjusted and weighted models, witnessing household SGBV (theorised mediator) at three months was associated with 3 times the odds of experiencing mild+ CMD symptoms (OR = 3.07; 95% CI [1.35–6.96]; *p* = 0.007). However, food insecurity as a potential baseline exposure was not significantly associated with probable mild+ CMD at six months (OR = 1.00; 95% CI [0.63–1.59]; *p* = 0.99). When the total effect was decomposed into the ADE, and ACME, we found that both the direct and indirect effects of food insecurity were very small and not statistically significant (ADE = –0.05; 95% CI [−0.16–0.07]; ACME = 0.02; 95% CI [−0.00–0.05]). The resulting proportion mediated by witnessing household SGBV was effectively 0%, reflecting the absence of the total effect of food insecurity on CMD. As indicated in Table 2 (models A and B), results were similar when looking at the relationship with probable moderate+ CMD. Restricting to participants without baseline household SGBV in the sensitivity analysis produced results consistent with the baseline-controlled models (Supplementary Table S2).

### Model 2: Mediating role of food insecurity in the association between witnessing household SGBV and CMD

In the final adjusted and weighted model, there was a significant overall association between witnessing household SGBV at baseline and probable CMD at six months, such that those exposed to SGBV had more than 2 times the odds of CMD than those not exposed to SGBV (OR = 2.19; 95% CI [1.26–3.81]; *p* = 0.006). As indicated in Table 3 (models A and B) there was also a significant association between household SGBV at baseline and the mediator of food insecurity at three months. Those exposed to SGBV had 50% higher odds of experiencing food insecurity than those not exposed to SGBV (OR = 1.55; 95% CI [1.06–2.27]; *p* = 0.024). When the total effect was decomposed into the ADE, and ACME, we found that most of the effect on probable CMD occurred directly through household SGBV exposure (ADE = 0.17; 95% CI [0.05–0.30]) rather than through the mediator of food insecurity (ACME = 0.01; 95% CI [−0.01–0.03]). The resulting proportion mediated by food insecurity was 5%. Results for moderate+ CMD followed a similar pattern, with a strong direct effect of household SGBV (ADE = 0.12; 95% CI [0.02–0.23]) and a very small indirect effect via food insecurity (ACME = 0.00; 95% CI [−0.01–0.02]), corresponding to 3% mediated. Restricting to participants without baseline food insecurity in the sensitivity analysis yielded results consistent with the primary baseline-controlled models. Specifically, the direct effect of SGBV on CMD remained strong, and the indirect effect via food insecurity was negligible (Supplementary Table S3).

## Discussion

Youth experience of food insecurity, SGBV, and symptoms of mental disorder are a growing concern in our post-COVID-19 world, where ongoing and future crises threaten to worsen exposure to adverse life events. This multi-directional mediation analysis contributes to understanding the longitudinal relationship between witnessing SGBV, food insecurity, and experience of common mental disorder among youth in low- and middle-income countries to identify effective intervention targets. Contrary to our primary hypothesis, witnessing household SGBV did not mediate an association between food insecurity and experience of CMD six months later among youth. Yet witnessing SGBV in one’s household at baseline was associated with later symptoms of CMD, with only a small mediating role of food insecurity, suggesting the ongoing importance of intervening on SGBV as a driver of CMD.

The relationship between food insecurity, exposure to SGBV, and CMD is understandably complex and most likely multi-directional. While most cross-sectional research testing associations between food insecurity and SGBV, and between SGBV and CMD would suggest that food insecurity precedes exposure to CMD (Purmotabbed et al., 2020), our results did not support this. It may be that food insecurity simply has a more acute impact on CMD, or that the results were influenced by the timing and context of data collection, which occurred shortly after a strict COVID-19 lockdown in South Africa. For example, it is possible that government, non-profit, and community efforts such as the Community Action Networks (CANs) (University of Cape Town, n.d.) to address food insecurity during this time limited the association with later CMD. Although testing the mediating role of food insecurity between witnessing household SGBV and subsequent CMD is not the usually theorised sequence of events, it is similar to other work, which has examined and found that housing insecurity mediates the relationship between witnessing SGBV towards one’s mother and later mental ill health among adolescents (Marçal, 2022).

Furthermore, the small mediating role of food insecurity in this study does not lessen the importance of addressing this valid health and human rights issue on its own. Structural interventions, such as cash transfers, which can decrease food insecurity, have been shown in recent meta-analyses to improve well-being and mental health among young people in low- and middle-income country settings (McGuire et al., 2022; Zimmerman et al., 2021). There is some evidence that cash transfer programs may also improve SGBV outcomes; however, this has mainly focused on IPV (Hidrobo et al., 2016; Kilburn et al., 2018).

There is a growing body of research on SGBV prevention interventions for youth across low- and middle-income countries, as highlighted by a 2015 systematic review of interventions (Lundgren & Amin, 2015). This review identified promising evidence around parenting and economic interventions, as well as community and school-based interventions targeting inequitable gender norms, healthy relationships, communication, and conflict negotiation skills. Many of these intervention targets may similarly improve youth mental health via SGBV reduction or on their own. However, future SGBV intervention research should measure and report on youth mental health as a secondary outcome to increase our understanding of which interventions may improve both outcomes.

### Limitations and future recommendations

There are several limitations of this study, including the relatively small sample size of 534 young people, which was greatly reduced at six months, resulting in low statistical power. In addition, the use of a single item to measure food insecurity on worries about having enough to eat does not allow for a comprehensive understanding of food insecurity in this population. Typical food insecurity measures, such as the Household Food Insecurity Access Scale (Coates et al., 2007), from which our item comes, includes nine items. While a longer scale would have allowed for the more precise measurement of the severity of food insecurity, we were limited by the overall survey tool length and needed to compromise to include many other measures required to respond to the broader study aims. Finally, there are potential unmeasured confounders such as household relationship dynamics (e.g., communication, conflict negotiation), as well as indicators from parents/guardians/other adult household members (e.g., their depression status). Given these limitations, future studies should continue to examine the mediating role of food insecurity, as well as other factors, which may mediate the impact of violence on youth mental health. These studies may consider using more robust measures of food insecurity, measuring outcomes from other household members, and testing the relationship with other forms of violence, such as intimate partner violence. Nonetheless, to the best of our knowledge, this is among the first studies to use mediation analysis to assess the direction and relative contribution of the relationships between food insecurity, witnessing household SGBV, and CMD, thus enhancing our understanding of these relationships.

## Conclusion

Our findings underscore the need to prevent and respond to household violence as a means of improving youth mental health. Witnessing SGBV in one’s household showed the strongest relationship with youth CMD symptoms over time in this bidirectional mediation analysis, compared to food insecurity. However, high prevalence of witnessing SGBV (33%), food insecurity (47%), and CMD symptomology (52%) highlights the urgent need for targeted interventions addressing all three adverse experiences. Future research should test these associations in larger studies and with other forms of SGBV, such as direct experiences of IPV. Testing the role of other potential drivers mediating the relationship between SGBV and youth mental health, such as alcohol use or other coping mechanisms, may help further refine our understanding of critical prevention intervention components.

## Supplementary Material

Suppl material

**Online supplementary material**: Supplementary data for this article are available at https://doi.org/10.2989/17280583.2025.2579960

## Figures and Tables

**Table 1.** Baseline participant characteristics, overall and by baseline exposure to household SGBV and food insecurity

	Total	Household SGBV, baseline			Food insecurity, baseline		
		
Baseline variables	*n*(%) 534	Yes *n*(%) 166	No *n*(%) 339	*p*-value	Yes *n*(%) 274	No *n*(%) 257	*p*-value

Current age, mean (SD)	19.0 (3.0)	19.4 (2.9)	18.9 (3.1)	0.06± 0.13	19.5 (3.1)	18.6 (2.9)	< 0.001***
Sex							0.013*
Women	373 (69.9%)	115 (69.3%)	239 (70.5%)		207 (75.5%)	164 (63.8%)	
Men	159 (29.8%)	49 (29.5%)	100 (29.5%)		66 (24.1%)	92 (35.8%)	
HIV status				0.006**			< 0.001***
YPLWH	214 (40.1%)	50 (30.1%)	145 (42.8%)		79 (28.8%)	134 (52.1%)	
YPLWoH	320 (59.9%)	116 (69.9%)	194 (57.2%)		195 (71.2%)	123 (47.9%)	
Household type				0.90			0.15
Formal dwelling	331 (62.0%)	100 (60.2%)	210 (61.9%)		159 (58.0%)	169 (65.8%)	
Informal dwelling	198 (37.1%)	64 (38.6%)	126 (37.2%)		111 (40.5%)	87 (33.9%)	
No. of people in household, mean (SD)	4.2 (2.6)	4.1 (2.8)	4.2 (2.5)	0.77	4.2 (2.6)	4.2 (2.6)	0.74
Had a sexual partner during lockdown	261 (49.7%)	89 (53.6%)	166 (49.0%)	0.33	149 (55.4%)	112 (44.1%)	0.010*
NEET	168 (31.8%)	69 (41.6%)	93 (27.8%)	0.002**	107 (39.5%)	60 (23.4%)	< 0.001***
Food insecurity	274 (51.6%)	104 (62.7%)	156 (46.3%)	< 0.001***	-	-	-
Food parcel receipt	171 (32.1%)	60 (36.1%)	103 (30.5%)	0.20	95 (34.8%)	75 (29.2%)	0.17
Probable mild+ depression (≥ 5)	238 (45.2%)	104 (63.0%)	120 (36.1%)	< 0.001***	134 (50.6%)	101 (40.2%)	0.019*
Probable moderate+ depression (≥ 10)	81 (15.6%)	44 (26.7%)	33 (9.9%)	< 0.001***	48 (18.1%)	33 (13.1%)	0.12
Probable anxiety	65 (12.4%)	27 (16.3%)	36 (10.7%)	0.073	34 (12.6%)	31 (12.3%)	0.91
Probable mild+ CMD	245 (47.3%)	108 (65.5%)	126 (38.0%)	< 0.001***	139 (52.3%)	106 (42.4%)	0.025*
Probable moderate+ CMD	116 (22.4%)	56 (33.9%)	54 (16.3%)	< 0.001***	66 (24.8%)	50 (20.0%)	0.19
Witnessing any household violence	166 (32.9%)	-	-	-	104 (40.0%)	62 (25.5%)	< 0.001***

*Note*. 29 participants were missing responses to exposure to household GBV, 3 were missing food insecurity at baseline, and 1 was missing household type. SD = standard deviation; *p*-values based on *t*-test and Pearson’s chi-square test. YPLWH = young people living with HIV; YPLWoH = young people living without HIV. NEET = not in education, employment, or training and includes participants ≥ 18 who are not in school and those < 18 who are not employed. Two participants (0.4%) selected “other” gender, and 4 (0.8%) selected “other” household type. Probable CMD is defined as a score of ≥ 5 on the PHQ-9 (range from 1–27) or scores ≥ 3 on GAD-2.

*** *p* < 0.001,

** *p* < 0.01,

* *p* < 0.05.

**Table 2.** Unadjusted and adjusted estimates of the mediation analysis testing the mediating role of witnessing household SGBV between food insecurity and probable (A) mild+ or (B) moderate+ CMD

	Model	Total effect	ADE	ACME	% mediated

A	Unadjusted	−0.04 (−0.16–0.07)	−0.05 (−0.17–0.06)	0.01 (−0.00–0.03)	0%
	Adjusted but not weighted for LTFU	−0.03 (−0.15–0.08)	−0.05 (−0.16–0.06)	0.02 (−0.00–0.05)	−0.3%
	Adjusted and Weighted for LTFU	−0.03 (−0.14–0.09)	−0.05 (−0.16–0.07)	0.02 (−0.00–0.05)	−0.2%
B	Unadjusted	−0.07 (−0.17–0.02)	−0.08 (−0.19–0.01)	0.01 (−0.00–0.04)	0%
	Adjusted but not weighted for LTFU	−0.05 (−0.14–0.05)	−0.07 (−0.16–0.02)	0.02 (0.00–0.06)	0%
	Adjusted and Weighted for LTFU	−0.07 (−0.16–0.02)	−0.09 (−0.18–0.00)	0.02 (−0.00–0.05)	0%

*Note*. Model A includes mild+ CMD. Model B includes moderate+ CMD. The adjusted model included baseline variables of age, gender, HIV status, household type, NEET status, IPV exposure, hazardous alcohol use, and self-isolation and an exposure to mediator interaction term. All models were adjusted for baseline values of the mediator and outcome. ACME = average causal mediated effect; ADE = average direct effect

**Table 3.** Unadjusted and adjusted estimates of the mediation analysis testing the mediating role of food insecurity in the association between witnessing household SGBV exposure and probable (A) mild+ or (B) moderate+ CMD

	Model	Total effect	ADE	ACME	% mediated

A	Unadjusted	0.17 (0.05, 0.30)	0.17 (0.04, 0.29)	0.01 (−0.00, 0.03)	5%
	Adjusted but not weighted for LTFU	0.18 (0.06, 0.29)	0.17 (0.06, 0.29)	0.01 (−0.01,0.03)	5%
	Adjusted and Weighted for LTFU	0.18 (0.06, 0.30)	0.17 (0.05, 0.30)	0.01 (−0.01,0.03)	5%
B	Unadjusted	0.12 (0.02, 0.23)	0.12 (0.02, 0.22)	0.00 (−0.01,0.02)	3%
	Adjusted but not weighted for LTFU	0.11 (0.01, 0.21)	0.11 (0.01, 0.20)	0.01 (−0.00, 0.02)	6%
	Adjusted and Weighted for LTFU	0.12 (0.03, 0.23)	0.12 (0.02, 0.23)	0.00 (−0.01, 0.02)	3%

*Note*. Model A includes mild+ CMD. Model B includes moderate+ CMD. The adjusted model included baseline variables of age, gender, HIV status, household type, NEET status, IPV exposure, hazardous alcohol use, and self-isolation and an exposure to mediator interaction term. All models were adjusted for baseline values of the mediator and outcome. ACME = average causal mediated effect; ADE = average direct effect
