# Supplementary material for: Interconnected effects of witnessing household violence, food insecurity, and mental health among peri-urban Cape Town youth: A mediation analysis
Source: J Child Adolesc Ment Health. Author manuscript; Available in PMC 2026 Mar 27. (PMC13018170; doi:10.2989/17280583.2025.2579960)
Supplement: Suppl material [file NIHMS2147478-supplement-Suppl_material.pdf]

Supplementary Information

Interconnected effects of witnessing household violence, food insecurity, and mental health among peri-urban Cape Town youth: A mediation analysis

Miriam Hartmann, Marie C.D. Stoner, Danielle Giovenco, Anna Mia Ekström, Abigail M Hatcher, Yanga Zembe Zondi, Nontembeko Qwabe, Audrey Pettifor, Linda-Gail Bekker, and Anna E. Kågesten

https://doi.org/10.2989/17280583.2025.2579960

Table S1. Comparison of baseline characteristics of the total unweighted sample with the weighted sample that completed all study visits

	Total - unweighted sample<br>%	All visits complete - weighted sample<br>%
**HIV status**		
YPWHIV	40	40
YPWoHIV	60	60
Total	100	100
**Gender**		0
Girl/woman / Intombi/umama	70	68
Boy/man / Ikwekwe/indoda	30	32
Other / Okunye	0	0
Total	100	100
**Household type**		0
Formal dwelling (a house, traditional hut, flat, townhouse, or room in a property	62	62
Informal dwelling (a shack or tent) / Ityotyombe (umkhukhu okanye umqasho)	37	37
Other / Okunye	1	1
Total	100	100
**In a sexual relationship during lockdown**		0
No / Hayi	50	50
Yes / Ewe	50	50
Total	100	100
**Food insecurity**		0
No / Hayi	48	49
Yes / Ewe	52	51
Total	100	100
**Receipt of food parcel**		0
No / Hayi	68	68
Yes / Ewe	32	32
Total	100	100
**Not in education or employment**		0
No	68	68
Yes	32	32
Total	100	100
**Any household violence, baseline**		0
No	67	69
Yes	33	31
Total	100	100
**Any IPV, baseline, including non-partnered**		0
No	91	91
Yes	9	9
Total	100	100
		0

	Total - unweighte d sample	All visits complete - weighted sample
	%	%
**Hazardous drinking (AUDIT-C), baseline**		
No	66	65
Yes	34	35
Total	100	100

Table S2. Estimates of the mediation analysis testing the mediating role of witnessing household SGBV between food insecurity and probable mild+ CMD

Model	*n*	Total effect	ADE	ACME	% mediated
Unadjusted	142	0.01 (–0.13, 0.14)	0.01 (-0.13, 0.15)	0.00 (-0.01, 0.02)	0.02%
Adjusted but not weighted for LTFU	141	0.00 (–0.12, 0.13)	0.00 (-0.13, 0.14)	0.00 (-0.02, 0.03)	0.01%
Adjusted and Weighted for LTFU	140	–0.01 (–0.15, 0.12)	–0.01 -0.14, 0.12)	0.00 (-0.02, 0.04)	-0.02%

Table S3. Estimates of the mediation analysis testing the mediating role of food insecurity in the association between witnessing household SGBV exposure and probable mild+ CMD

Model	N	Total effect	ADE	ACME	% mediated
Unadjusted	101	0.31 (0.10, 0.50)	0.31 (0.12, 0.50)	0.00 (-0.06, 0.07)	-0.18%
Adjusted but not weighted for LTFU	88	--	--	--	--
Adjusted and Weighted for LTFU	88				

Note. Adjusted models did not converge due to missing Y (estimation error)
